# New Pieces for the Older Americans Act Puzzle

**DOI:** 10.1093/geroni/igaa057.2574

**Published:** 2020-12-16

**Authors:** Andrew MacPherson

**Affiliations:** Healthsperien, Washington, District of Columbia, United States

## Abstract

With the passage of the new “Supporting Older Americans Act of 2020,” Congress acknowledged and address several under appreciated issues -the growing social isolation and loneliness of older adults, and the significant role that the aging network does and can play in providing supportive services to older adults with advanced illness (also known as serious illness). This presentation will provide information on these and other pieces of the OAA puzzle.

